# Effects of a DRG-based hospital reimbursement on the health care utilization and costs in Swiss primary care: A retrospective “quasi-experimental” analysis

**DOI:** 10.1371/journal.pone.0241179

**Published:** 2020-10-27

**Authors:** Omar Al-Khalil, Fabio Valeri, Oliver Senn, Thomas Rosemann, Stefania Di Gangi

**Affiliations:** 1 Institute of Primary Care, University and University Hospital of Zurich, Zurich, Switzerland; 2 Department of Infectious Diseases, Bern University Hospital, University of Bern, Switzerland; Flinders University, AUSTRALIA

## Abstract

**Introduction:**

In Switzerland, a nationwide Swiss Diagnosis related Groups (Swiss DRG) system for hospital reimbursement was introduced in 2012. However, the impact of DRG systems on primary care is still unclear with respect to number of consultations and costs. The aim of this study was to investigate the effect of the implementation of DRG on costs and volumes in the primary care sector, on a nationwide basis in Switzerland.

**Methods:**

The study retrospectively analysed yearly data, from 2008 to 2014, of almost 60 Swiss health insurers that covered almost all Swiss general practitioners, with a total number of patients which represented approximately 76% of the Swiss population. GP consultations, total numbers and rates, and the relative costs reimbursed (TARMED tariff values) in the Swiss federal states, cantons, which already introduced a DRG-like system before 2012 (AP-DRG), were compared to the GP consultations and costs reimbursed in the other cantons (DRG-naive). Regression discontinuity design analysis and mixed regression models, at cantonal level, were performed to evaluate the effect of the nationwide implementation of the Swiss DRG on health care demand and costs in the primary care setting. Change in outcome level and yearly trend pattern difference between groups (AP-DRG vs. DRG-naive) were examined.

**Results:**

Overall, the total number of GP consultations and the relative TARMED values increased from 2008 to 2014. In the DRG naive, 15 cantons: in 2008, the number of GP consultations were 13,114,126, with a TARMED value of 1,194,957,157 CHF, and in 2014, the GP consultation were 13,752,511, with a TARMED value of 1,513,861,260 CHF. In the AP-DRG group, 11 cantons, the total number of GP consultations increased from 8,787,646, in 2008, to 9,347,168 in 2014 and the TARMED value increased from 896,673,657 CHF in 2008, to 1,100,203,508 CHF in 2014. The yearly trend pattern of GP consultations and TARMED values, in the AP-DRG group, were not significantly different from the respective trends in the DRG- naive and, overall, no significant change was detected in consultations and costs trends before and after 2012.

**Discussion/Conclusion:**

This study found no evidence of any effect of the introduction of the SwissDRG on the yearly trend of primary care consultations and costs. Nevertheless, potential negative impacts on vulnerable patients, as chronically ill patients, could not be excluded and further investigation is required.

## Introduction

Since 1970, when the Organization for Economic Cooperation and Development (OECD) began recording worldwide health spending, total and by type of financing, the Swiss health sector has been characterized by a steady increase in total health expenditure, from a yearly rate of 4.9% of GDP, in 1970, to a yearly rate of 12.14% of GDP in 2019 [1]. Hospital sector has been a key driver of costs since, compared with other OECD countries, Switzerland has a high number of hospitals for its population and geographic size [2], 33 hospitals per million of inhabitants in 2018 [1]. Moreover, until 2012, Swiss hospitals were generally remunerated through daily rates or fee-for-services, though eleven Swiss federal states, or cantons, implemented a Diagnosis Related Groups (DRG) financing scheme, AP-DRG (All Patients Diagnosis-Related Groups), years before 2012 [3]. In 2012, the DRG system, called Swiss Diagnosis Related Groups (Swiss DRG), was implemented in the whole country, which now covers more than 8,500,000 people.

Other countries in Europe have already introduced the DRG system with the aim of improving transparency, increasing efficiency and reducing costs [4].

The DRG system might create false incentives because, in order to be economically efficient, hospitals could shorten the length of stay and the number of services provided, and at the same time, maximize the number of cases [5]. Moreover, hospitals could be encouraged to avoid high-cost patients by shifting them to other providers ("cost-shifting") or to discharge them inappropriately early ("bloody exits") [6].

In addition, an increase in the number of cases may have an impact on admission regulations. It may lead to more non-medically indicated treatments, to inpatient rather than outpatient treatment, or to a division of care episodes into multiple admissions. These different effects can be relevant for primary care, as shifting these patients can lead to more morbidity and thus to more cost-intensive treatments.

In USA, and also in most European countries, the quality of care had not been significantly affected by the DRG introduction [7]. In Korea, a decrease in length of hospital-stay and, on the other hand, an increase in outpatient visits were found as an effect of DRG introduction [8, 9]. In Switzerland, there was no evidence of a significant deterioration in patient care or a significant change in the length of stay (LOS) under SwissDRG [6, 10, 11] though an association with readmission rates was found [11, 12].

Previous research [10] gave no evidence of a shift in costs for primary care. Anyway, the analysis, based on data from two hospitals, was limited to a period of six months before and after the SwissDRG implementation.

Therefore, the aim of the current study is to better investigate the impact of SwissDRG on costs and volumes in the primary care sector, analyzing data, from 2008 to 2014, of all Swiss cantons.

## Materials and methods

### Data

The data used for this study were provided by SASIS AG Switzerland, a data warehouse company of Santésuisse, an umbrella organization of Swiss statutory health insurers. The dataset, from almost 60 health insurers, included on average 76% of the Swiss population. The SASIS AG collects aggregated administrative claims data for drug prescriptions (SASIS Tarifpool) and health care services data of licensed physicians (SASIS Datenpool) from patients insured in the statutory health system.

The dataset contained, in one file, the list of all Swiss ambulatory care providers and the area of their practice locations. Each provider was anonymously identified through a number (ZSR) and classified into the medical specialties, according to the Swiss Medical Association (FMH). In another file, the dataset contains, for each provider, the aggregated number of consultations and the total cost of reimbursement (including medical treatments) per year. Consultations and reimbursements were classified by patient’s gender, by patient’s age groups (5 years classes, from age 0 to 95) and by community of patient residence. For this study, only consultations provided by primary care physicians, in the period from 2008 to 2014, were considered. Moreover, accident-related consultations were excluded due to different health insurance coverage. The study frame was chosen according to the data availability at the time when our research started and in order to have at least two years of observations before and after the DRG introduction. In fact the first year after DRG introduction might be not representative due to the system change (learning curve). Therefore having a second year of follow up after introducing the novel system provided more robust data.

**Fig 1:**
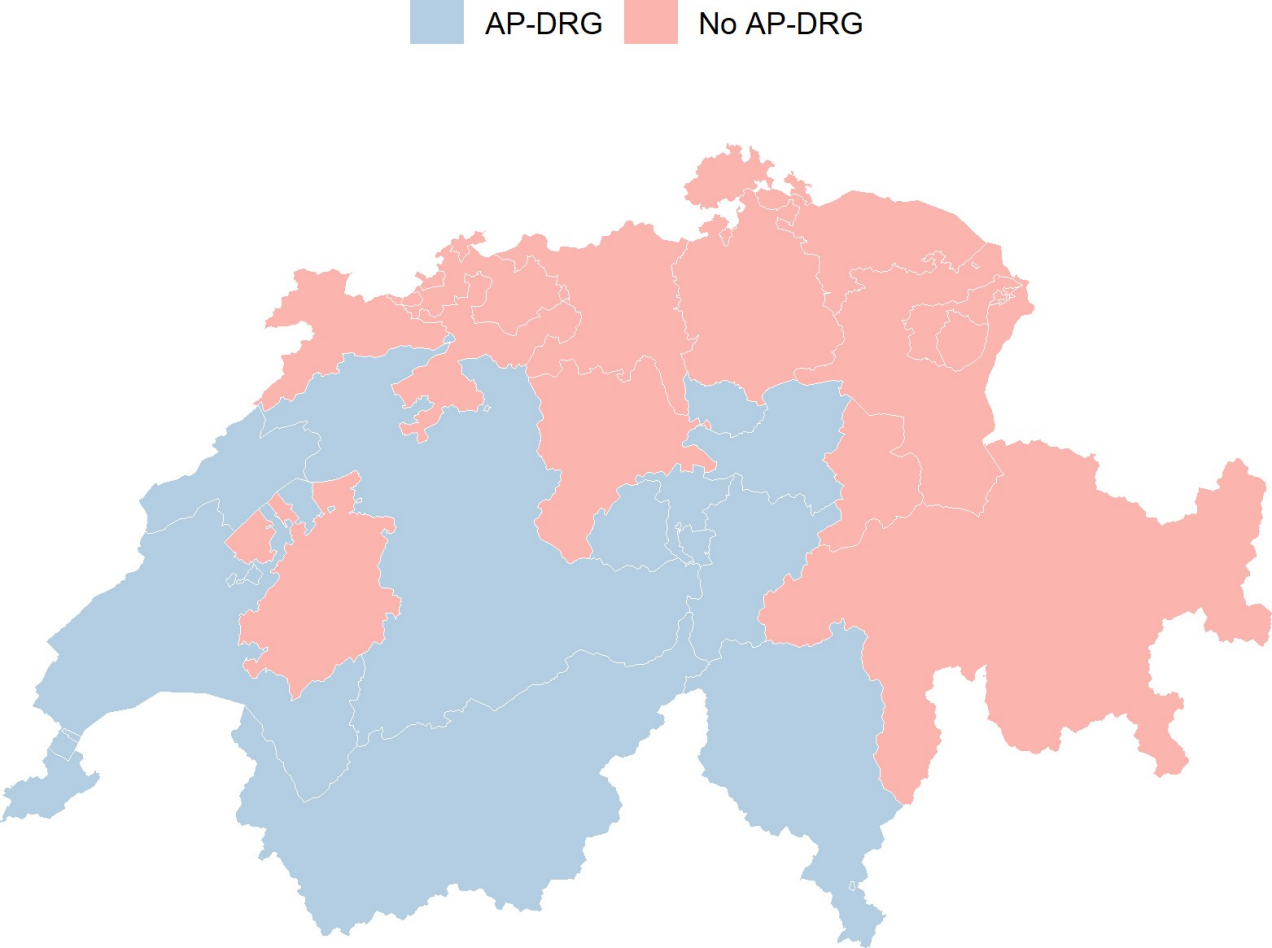
Swiss cantons by DRG-group.

SASIS data were grouped at cantonal level, 26 cantons, and then at DRG groups: DRG-naive (No AP-DRG) and AP-DRG (Fig 1).

In the primary analysis, we included only the consultations of patients living in the same canton of the GP. As a sensitivity analysis, we considered all the consultations at GP cantonal level. Overall 11 cantons already used AP-DRG before 2012: Bern (BE), Geneva (GE), Neuchâtel (NE), Nidwalden (NW), Obwalden (OW), Schwyz (SZ), Ticino (TI), Uri (UR), Vaud (VD), Valais (VS), Zug (ZG). The other 15 cantons introduced DRG after 2012: AG (Aargau), Appenzell I. Rh. (AI), Appenzell A. Rh. (AR), Basel-Landschaft (BL), Basel-Stadt (BS), Fribourg (FR), Glarus (GL), Graubünden (GR), Jura (JU), Lucerne (LU), St. Gallen (SG), Schaffhausen (SH), Solothurn (SO), Thurgau (TG), Zurich (ZH). Cantons in the AP-DRG group did not introduce AP-DRG simultaneously, but all cantons introduced it before 2008 [13,14]. Therefore, the AP-DRG group was consistent during the study period.

GP consultations were adjusted for the permanent resident population by canton, age-group, and sex. Data were collected from the Federal Statistics Office data (www.statistics.admin.ch). Therefore, we distinguished between the total (observed) GP consultations and the expected ones, which were computed adjusting the observed consultations with the yearly population change relative to the reference population in 2010. The gross costs for the GP consultations included all invoices that were reimbursed within the framework of compulsory health insurance, including the cost-sharing of the insured persons (franchise, deductible, contribution to the costs of hospital stays). Based on the TARMED reimbursement tariff values, cost weights were generated and introduced in 2004 for the regional usage of services provided by GP [15]. TARMED cost weights (or TARMED tariff values) were calculated by dividing claims in Swiss Francs (CHF) by a reimbursement factor for medical services, negotiated annually between medical associations, health insurers, and health authorities on a cantonal level. Per capita, utilizations: per resident person, in 2010, per patient and consultation were calculated based on these cost weights. The ratio of the cost-sharing of the insured persons versus TARMED cost weights was also calculated. Analogously to the GP consultations, the TARMED cost weights were adjusted for the permanent resident population by canton, age-group, and sex. Therefore, the expected TARMED tariff values were computed, adjusting the observed TARMED tariff values with the yearly population change relative to the reference population in 2010.

### Ethics approval

According to the national ethical and legal regulation, an ethical approval was not needed. License to access the study data was provided by SASIS AG. Since data were anonymised, no consent of patients was required. This study followed the Strengthening the Reporting of Observational Studies in Epidemiology (STROBE) reporting guideline.

### Study design

The study was retrospective with a quasi-experimental fuzzy design [16,17]. We have a regression discontinuity in time, or an interrupted time series data [18], where time was the running variable, with a treatment, or intervention, (e.g. DRG implementation) by the year 2012, as the threshold. The causal treatment, or intervention, effect could be estimated through the difference in the outcome variable in the two groups, treatment (DRG-naive) and control (AP-DRG) evaluated before and after 2012, the year of DRG introduction. The fuzzy design occurred since only a subgroup followed a treatment assignment rule; that is after 2012 only the DRG-naive were treated. Therefore, the introduction of the DRG was not a deterministic function of time, but the year 2012 could be seen as a threshold where the probability of the DRG intervention jumped.

### Statistical analysis

Yearly data were aggregated by DRG groups as described above (AP-DRG = Yes, 11 cantons; AP-DRG = No, DRG-naive, 15 cantons). Descriptive tables of GP consultations reported, for each year and group: the total number of GP consultations (observed and expected), the total population, the average number of GP consultations per canton (or the GP consultations at canton level), the rate of expected GP consultations, the number of GPs, the number of patients. The rate of expected GP consultations was defined as the total number of expected GP consultations divided by the total number of inhabitants in 2010, chosen as a reference population. We also reported the overall average percentage of the number of consultations where the patient’s canton of residence did not differ from the provider’s canton. Descriptive tables of the costs for GP consultations reported, for each year and group: the total gross costs, the TARMED tariff value, observed and expected, total and per capita (per person, patients and consultation), the total expected patient cost-sharing and the ratio cost-sharing versus Total expected TARMED. The TARMED tariff per person was computed, dividing the total expected TARMED value by the total number of inhabitants in 2010, chosen as the reference population. The TARMED per patient was computed, dividing the total expected TARMED value by the number of patients in each group/year. The TARMED per consultation was computed, dividing the total expected TARMED value by the total number of expected GP consultations. The total expected patient cost-sharing was computed, adjusting the observed cost-sharing for the permanent resident population by canton, age-group, and sex. An additional descriptive table for GP consultations and costs, stratified by the patient’s sex and age group, was reported as S1 Table.

Moreover, to evaluate the cantonal disparities, in terms of GP consultations and costs, we calculate the Gini coefficient, overall, within DRG groups and by years. The Gini coefficient is a measure of inequality in a distribution. It ranges from 0, perfect equality, and 1, perfect inequality [19].

We performed mixed models with random effects at canton level to identify the trend pattern, of the GP consultations and the relative TARMED tariff in the two DRG groups, corrected for canton’s correlation from 2008 to 2014. DRG area was a fixed effect, alone and with interaction with time. A quadratic growth over time (Time^2^+Time) was detected. An autocorrelation of order 1, AR(1), described the within-group correlation structure. Time was scaled defining calendar year 2012 as time 0. In details, the model was specified as follows:

$$\begin{array}{l} Y∼intercept+[Fixed\;effects=\left(Time^{2}+Time\right)+AP‐DRG\;group+\\ \;\left(Time^{2}+Time\right):\;AP‐DRG\;group+x_{2}]+[Random\;effect=canton] \end{array}$$

where (Time^2^+Time): AP-DRG group denoted the interaction between time and AP-DRG group; x_2_ was another predictor, in dependence of the specific outcome.

The outcomes, Y, were: a) GP consultations expected at canton level; b) rate of expected GP consultations at canton level; c) the expected TARMED tariff values of GP consultations per capita (per person). The interaction term was only considered for outcome a), since the fit for the other outcomes, was better without it. The other predictor x_2_ was the number of GP for outcome a), the rate of GP (GP/Population at time 2010)×10,000 inhabitants, for outcome b), and the rate of expected GP consultation for outcome c). The results of these statistical models were reported as estimates (standard errors). The autocorrelation structure coefficient ɸ was also reported. For outcome a), random effects at both coefficient and slope were used since the fit was better. For the other outcomes, random intercept models were sufficient. For outcome b) and c), an additional analysis, including as covariate patient’s sex and age group, was performed, and results were shown graphically as S1 Fig.

Regression discontinuity design analysis allowed a visual comparison of the time series pattern, before the DRG introduction, with the pattern after the DRG introduction and the assessment of the change in outcome level, after the DRG intervention, in relation to the pre-introduction pattern. Local linear regressions were performed for outcome b) and c) in the neighborhood of the threshold, 2012. Predictors were time, DRG-naive group indicator, the interaction term (time: DRG), and the additional covariate x_2_ for outcome b) and c) respectively. Models were also corrected for correlated observations at the canton level. The “neighborhood” of the threshold was defined using weights based on a quartic kernel and an “optimal” bandwidth as defined in [20]. For an intuitive presentation of these models, we showed results only visually.

All tests with P < 0.05 were considered statistically significant. All analyses were carried out using statistical software R (https://www.R-project.org). In particular, for the regression discontinuity analysis, we used R package rdd.

## Results

### Descriptive statistics

In Table 1 we reported the GP consultations (observed and expected), the rate of expected GP consultations, and the demographic characteristics of cantons by DRG groups.

**Table 1:** GP consultations and demographic characteristics of cantons by DRG groups.

Year	AP-DRG	Total GP Consultations (Observed)*	Total GP Consultations (Expected)	Total Population	GP Consultations at canton level (mean per canton)	Rate of expected GP consultations	Number of GP	Number of Patients
2008	No	13,114,126	13,514,496	4,423,223	874,275	2.98	3704	3,245,308
2008	Yes	8,787,646	9,069,437	3,262,019	798,877	2.71	3144	2,191,284
2009	No	13,003,076	13,202,226	4,474,721	866,872	2.91	3745	3,291,528
2009	Yes	8,752,835	8,898,288	3,299,053	795,712	2.66	3175	2,222,252
2010	No	13,034,052	13,034,052	4,529,598	868,937	2.88	3831	3,408,959
2010	Yes	8,898,772	8,898,772	3,341,983	808,979	2.66	3231	2,277,371
2011	No	13,072,219	12,882,920	4,581,050	871,481	2.84	3921	3,498,973
2011	Yes	8,795,917	8,674,814	3,374,988	799,629	2.60	3302	2,340,248
2012	No	13,161,802	12,786,713	4,631,856	877,453	2.82	4023	3,577,940
2012	Yes	8,858,915	8,612,361	3,408,555	805,356	2.58	3406	2,374,829
2013	No	13,882,635	13,295,635	4,686,368	925,509	2.94	4108	3,876,695
2013	Yes	9,301,225	8,899,227	3,454,488	845,566	2.66	3521	2,497,368
2014	No	13,752,511	12,978,062	4,742,717	916,834	2.87	4181	3,882,785
2014	Yes	9,347,168	8,803,229	3,496,121	849,743	2.63	3639	2,515,008

Two DRG groups were identified: 11 cantons which used AP-DRG (BE, GE, NE, NW, OW, SZ, TI, UR, VD, VS, ZG) and 15 cantons which introduced DRG after 2012 (in details, we have AG, AI, AR, BL, BS, FR, GL, GR, JU, LU, SG, SH, SO, TG, ZH). The rate of expected GP consultation was defined as the total GP Consultations (Expected) / Total Population in 2010.

*On average, in 94% of all patient consultations there was concordance between patient residency (e.g. canton level) and GP location.

The analysis showed that in 94% of all patient consultations there was concordance between patient residency (e.g. canton level) and GP location. Overall, in the DRG-naive group, the sum of GP consultations (observed) increased from 13,114,126 in 2008 to 13,752,511 in 2014. Instead, the sum of GP consultations (expected) decreased from 13,514,496 in 2008 to 12,978,062 in 2014. Analogously, the rate of expected GP consultation, per inhabitant, decreased at the same rate, from 2.98 in 2008 to 2.87 in 2014. The number of patients increased more compared to the number of GP (yearly average of growth of 3.03% vs 2.04%). In the AP-DRG area, the sum of GP consultations (observed) increased more compared to DRG-naive (yearly average of growth 1.03% vs 0.79%). The total of GP consultations (expected), instead, decreased less, compared to DRG-naive (yearly rate of growth of -0.49% vs. -0.67%) with 2.71 consultations per inhabitant. Differently from the DRG-naive, the number of GP increased more than the number of patients (yearly rate of growth of 2.47% for GP compared to a rate of growth of 2.32% for the number of patients). We also observed that the ratio number of patients / number of GP was higher in the DRG-naive group compared to the AP-DRG group.

As regarding the inequality, between cantons, in the distribution of the total GP consultations, weighted for the total population, we found an overall Gini coefficient of 0.40, which remained approximately unchanged during years 2008–2014. Within the groups, we found that the Gini coefficient in AP-DRG decreased from 0.35, in 2008, to 0.32 in 2014. In DRG-naive, instead increased from 0.40, in 2008, to 0.41 in 2014.

In Table 2, we reported the costs of the GP consultations.

**Table 2:** Costs of GP consultations by DRG groups.

Year	AP-DRG	Total gross costs (CHF)	TARMED tariff values (CHF) of GP consultations					Patient cost-sharing	Ratio (b)/(a)
		observed	Total observed	Total expected (a)	Per person(exp)	Per patient (exp)	Per cons. (exp)	Total expected (b)	
2008	No	1,033,802,791	1,194,957,157	1,232,028,426	278.54	379.63	91.16	295,031,186	0.24
2008	Yes	808,987,625	896,673,657	926,230,867	283.94	422.69	102.13	197,763,616	0.21
2009	No	1,052,663,923	1,215,981,007	1,234,928,262	275.98	375.18	93.54	275,137,331	0.22
2009	Yes	818,001,421	908,110,753	923,683,617	279.98	415.65	103.80	190,368,471	0.21
2010	No	1,069,789,748	1,234,935,134	1,234,935,134	272.64	362.26	94.75	272,464,090	0.22
2010	Yes	834,166,336	925,536,753	925,536,753	276.94	406.41	104.01	187,771,964	0.20
2011	No	1,113,170,879	1,282,725,304	1,263,857,915	275.89	361.21	98.10	282,703,775	0.22
2011	Yes	858,712,824	952,714,607	939,343,182	278.32	401.39	108.28	193,065,309	0.21
2012	No	1,152,184,860	1,326,852,153	1,288,529,212	278.19	360.13	100.77	299,799,366	0.23
2012	Yes	888,128,440	987,304,470	959,473,683	281.49	404.02	111.41	205,777,687	0.21
2013	No	1,252,157,168	1,442,374,897	1,380,623,625	294.60	356.13	103.84	323,745,885	0.23
2013	Yes	949,029,295	1,060,182,149	1,013,514,187	293.39	405.83	113.89	217,475,689	0.21
2014	No	1,318,154,823	1,513,861,260	1,427,652,061	301.02	367.69	110.01	327,354,480	0.23
2014	Yes	985,663,061	1,100,203,508	1,034,662,063	278.54	379.63	91.16	220,266,528	0.21

Values reported in CHF: total gross costs, TARMED tariff values (total, per person, per patient, per consultation = per cons.) and patient cost-sharing. In the last column, the ratio patient cost-sharing / TARMED expected total value was reported.

Overall, the total gross costs increased from 2008 to 2014 in both groups, with a yearly growth rate of 4.13%, in the DRG-naive group, compared to a yearly rate growth rate of 3.35% in the AP-DRG area. The total TARMED tariff values, observed and expected, also increased in both groups and the yearly growth was higher in the DRG-naive group compared to the AP-DRG group. The per-capita TARMED expected values were higher in the AP-DRG group. The tariff values per person were increasing in both groups at, approximately, the same rate. The tariff values per patient, instead, were decreasing in both groups. The tariff values per consultation, instead, increased from 2008 to 2014 in both groups with a greater yearly rate in the DRG-naive group compared to the AP-DRG group (3.18% vs. 2.37%).

Additional details on the number of GP consultations and TARMED positions, stratified by sex and age group of the patient, were provided in the S1 Table. We observed that the consultation rate for older patients is on average 6 times the consultation rate for younger patients, in the AP-DRG group, and 5 times in the DRG-naive group.

As regarding the inequality, between cantons, in the distribution of the TARMED positions, weighted for the total population, we found an overall Gini coefficient of 0.39, in 2008 and of 0.41 in 2014. Within the groups, the Gini coefficient in AP-DRG was of 0.32, unchanged from 2008 to 2014. In DRG-naive, instead increased from 0.41, in 2008, to 0.43 in 2014.

### Mixed models

In Table 3, we reported the results of the mixed models for the GP consultations (expected) and the rate of expected GP consultations at canton level.

**Table 3:** Total GP expected consultations, rate of GP expected consultations and TARMED tariff (expected, per person) at canton level.

	GP Consultations (expected)		TARMED tariff (expected)
	Total	Consultation rate	Per person
AP-DRG = 1	-161,612.70 (122,695.10)	-0.164 (0.151)	21.919 (11.651)
Time^2^	2,000.17** (763.658)	0.007* (0.003)	0.860*** (0.129)
Time	-12,910.77** (4,876.62)	-0.020 (0.012)	8.262*** (0.496)
Number of GP	2,224.40*** (126.38)		
	
AP-DRG = 1:Time^2^	-1,840.84 (1,176.99)		
	
AP-DRG = 1:Time	-7,352.81 (7,425.69)		
	
Rate of GP's × 10'000 inhabitants		0.022 (0.029)	
Intercept	267,219.40** (86,581.18)	2.600*** (0.276)	53.713*** (11.623)
Consultation rate			77.122*** (3.134)
N	182	182	182
Autocorrelation ɸ	0.37	0.73	0.98

*Note*:

*p<0.05

**p<0.01

***p<0.001

Mixed models with fixed effects (interaction term AP-DRG and time, with quadratic trend), random effects (canton) and autocorrelation. For each effect, estimates and standard errors (within parentheses) were reported. Moreover, ɸ, autocorrelation structure coefficient, for each model was shown.

After correcting for measurement correlation at canton level, the difference between the DRG groups was not due to the introduction of the nationwide DRG financing system. There was no significant effect of AP-DRG group, alone, or in interaction with time. The expected GP consultations significantly decreased over time in both groups, according to a quadratic trend. Any additional GP, had an effect of 2,224 (se = 126.38, p<0.001) GP yearly consultations, at canton level. The autocorrelation within groups was ɸ = 0.37.

Analogously, for the rate of expected GP consultations the trend was decreasing in both groups, according to a quadratic trend statistically significant. There was no significant effect of AP-DRG group and of the rate of the number of GP over the population. The autocorrelation within groups was ɸ = 0.73.

Instead, the TARMED tariff per patient, in both groups, was increasing, according to a quadratic trend, statistically significant. The costs per capita were higher in the AP-DRG area but we did not find a statistically significant effect due to the introduction of the DRG. Each additional consultation, per person, or consultation rate, had an effect of near 77 CHF in the TARMED tariff pro capita. The autocorrelation within groups was ɸ = 0.98.

**Fig 2:**
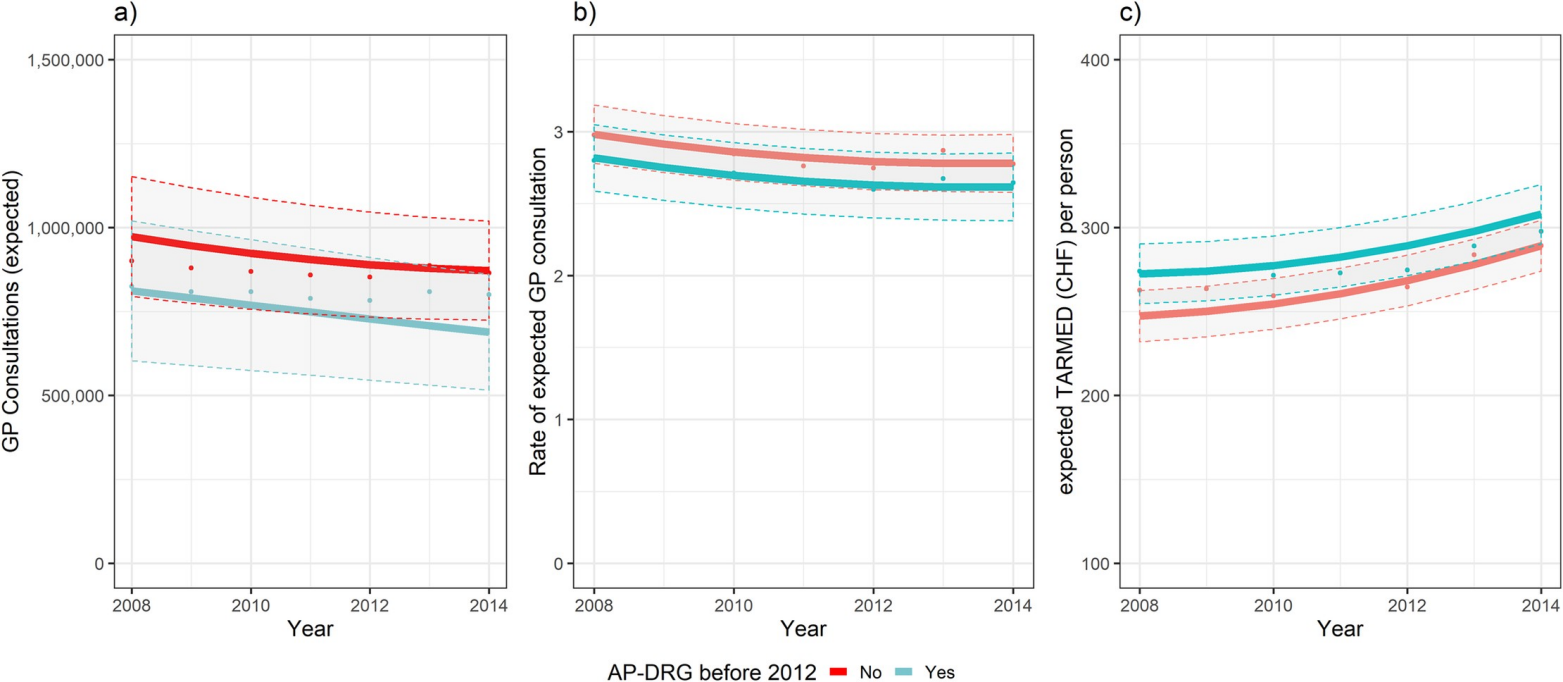
Effects of DRG over time on: a) GP consultations (expected) at canton level; b) Rate of expected GP consultations; c) TARMED tariff (expected) per person for GP consultations at canton level. Points were averages of observed values. Lines were fitted values from the mixed models, marginal effects. Dotted lines were borders of 95% confidence bands for the two groups.

In Fig 2, we represented graphically the trend for a) the expected GP consultations b) the rate of expected GP consultations, or consultation rate c) the expected TARMED tariff value per person.

Points were yearly averages, at canton level, of the observed values in the two groups. Lines were fitted values, marginal effects, from the mixed models shown in Table 3. Dotted lines are borders of 95% confidence bands for the two groups. As sensitivity analysis, we represented, S1 Fig, the plot of the mixed model for outcome a) and b) adding as covariate the age group and sex of the patient. We observed a significant difference in outcome b) between the two groups: AP-DRG and DRG-naive group but only for older patients. However, we found no significant effect of the main term overall (AP-DRG vs. DRG-naive).

### Regression discontinuity analysis

**Fig 3:**
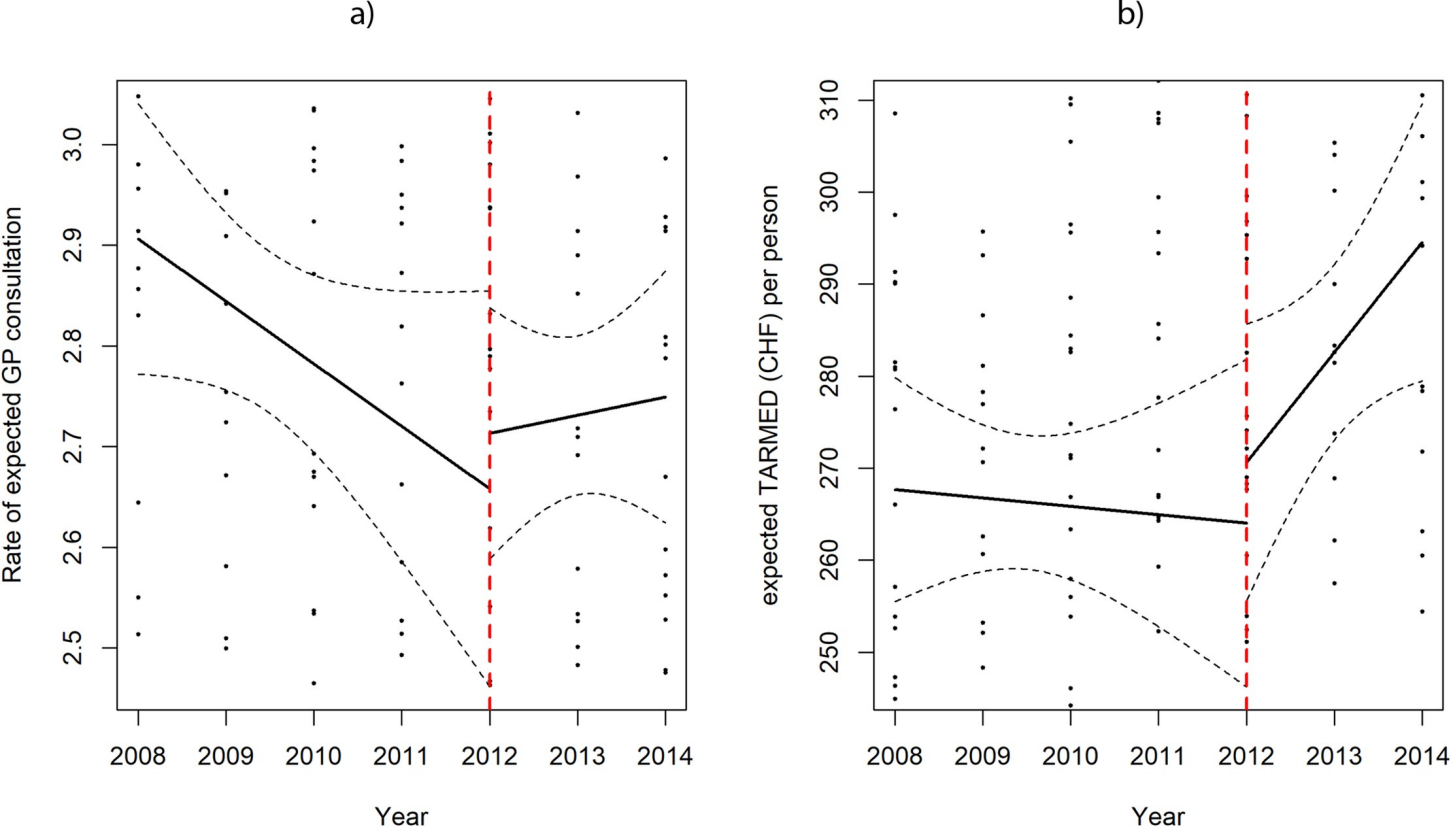
Regression discontinuity, fuzzy design, plot for: outcome a) Rate of expected GP consultations, or GP consultation rate; outcome b) TARMED tariff (expected) per person for GP consultations at canton level. Local linear regressions, with optimal bandwidths, were plotted to either side of year 2012, when DRG was introduced.

In Fig 3 the local regression analysis with the optimal bandwidth plot was represented for outcome a) and b). Since bandwidths, to either side of 2012, were overlapping, no evidence of effect of the introduction of DRG on the outcomes could be shown.

## Discussion

In this study, we investigated the impact of the introduction of the SwissDRG on GP consultations and their relative costs. Based on data provided by GPs, we used a quasi-experimental approach with an interrupted time series, comparing cantons using AP-DRG before the implementation of SwissDRG with DRG-naive cantons after implementation.

The implementation of a DRG-based payment system is a well-established way to create transparency of costs and to help to reduce health-related expenditures [21, 22]. Most research has focused on (inpatient) care in hospitals rather than outpatient care [11]. Accordingly, there is little data on primary care, especially about the outpatient care sector, which affect the consultations [4, 23–25]. Additionally, only a minority of articles presented empirical data, and most of them are not 'evidence-based' [26].

### Trends

When comparing the trends over 2008–2014, we found no difference regarding the sum and rate of expected GP consultations and expected reimbursement.

This finding supports the results of other Swiss studies, which showed no rise in GP-consultations [10, 12]. One study [12] was based on prediction from data collected before the introduction of SwissDRG and the other [10] focused only on data from two hospitals and was limited to a period of six months before and after the implementation of SwissDRG. Our study, instead, was much more extensive, since it covered claim data from almost 76% of the Swiss population, collected over six years, four years before and two years after the implementation of SwissDRG.

Moreover, we observed a significant non-linear decrease in GP consultations and a significant non-linear increase in costs in both groups, independent of the DRG system. These trends had the following explanations: in DRG-naive, from 2008 to 2014, the number of patients, and so the demand for primary care, increased more compared to the supply of primary care; in AP-DRG group, cantons were smaller and less populated, with fewer patients and, therefore, fewer consultations per inhabitant, meaning higher and increasing per-capita costs.

### Shift of cost weights for vulnerable patients

In order to investigate a possible "shift" of older and possibly more vulnerable patients to primary care, we also conducted a stratified patient analysis according to age and gender. We found no effect of the introduction of SwissDRG on GP consultations, changing with patient age, but a slight effect on the relative per-capita costs. However, for both results, this analysis did not indicate an overall difference in outcome between the two DRG-groups, as the main term was not significant. In fact, the trend was similar in both DRG-groups for each age class, but the levels of costs and consultations depended on age. This is in line with other studies: [27] showed that an increase in the number of the elderly and infants, in the British population, led to a higher consultation rate of these 'extreme' age groups and [28] showed that, in Switzerland, an increase in consultation rates was associated with increasing age. In fact, the demand for primary care consultations is likely due to the accumulation of chronic conditions in the aging population [29–31].

Moreover, an increase in costs covered by other payment systems, such as rehabilitation, transitional care, or medical home care could not be excluded. Swiss Acute and Transitional Care Act (ATC) was introduced, one year before the SwissDRG, to reduce and prevent possible adverse effects of the DRG reimbursement system, primarily with regard to vulnerable patient groups. Impact of ATC and its effects on discharge of patients, with persisting care needs after hospitalisation, were investigated in a study by Kone et al. [32]. Despite the introduction of ATC, a recent study [33] highlighted different results in different cantons leading to potential disadvantages for patients and calling for a need of improvement.

### Strengths/Limitations

Some limitations of our study have to be acknowledged. First, we used claim data only measuring age, gender and residency as patient characteristics and we did not include other relevant characteristics affecting GP consultations and costs. Second, we did not consider in our model, for the total of expected GP consultations, any other confounding factors related to cantonal disparities between the DRG groups (i.e., socio-economic characteristics). However, to face this limitation, we measured the inequality of GP consultations and costs, through the Gini-coefficient. The overall coefficients of 0.4, for both GP consultations and costs, weighted for the population, showed an almost high inequality. This came directly from the fact that almost 70% of the 26 cantons, represented only 40% of the total GP consultations and costs. Moreover, the indexes of inequality resulted slightly lower in the AP-DRG group, compared to the DRG- naive. However, the overall cantonal disparities remained unchanged from 2008 to 2014, and therefore we could exclude a possible influence of the DRG introduction.

Third, we have no data on inpatient-outpatient transitions regarding patients, which are not directly discharged due to the requirement of transitional care.

However, our study has several strengths. It provides a large sample size, with almost 76% of patients-data in primary care. Another strength is the period of overall six years, two years before and four years after SwissDRG-implementation. Furthermore, we used a strong study-design, which allowed us to describe temporal changes by using AP-DRG cantons as a control group. Finally, the statistical models were accurate, controlling for both random effects and autocorrelation. In fact, autocorrelation in consultation rate and per-capita costs resulted high and therefore relevant to be accounted for.

## Conclusions

The number of consultations of general practitioners (GP) at the canton level showed a decreasing trend. Instead, the relative costs per capita, showed an increasing trend. However, we could not give evidence of a 'cost shift' after the introduction of the SwissDRG in 2012. Detected structural and cantonal differences were independent of the fact that some cantons had already introduced the DRG in the form of AP-DRG before 2012. Future studies should evaluate the impact of DRG focusing on vulnerable patient groups and quality of care. Accurate information on inpatient-outpatient transitions is also required.

## Supporting information

S1 Checklist The RECORD statement–checklist of items, extended from the STROBE statement, that should be reported in observational studies using routinely collected health data. (DOCX)

S1 Table GP consultations and costs stratified by DRG-group, patient age groups (0–35, 36–65, 66–95) and sex. Total GP Cons. Exp., GP consultation expected; Rate of exp cons, consultation rate. TARMED position expected, Total and per Person, were reported by DRG groups.
(TIF)

S1 Fig Effects of DRG over time and for patient’s age groups on: a) Rate of expected GP consultations; b) TARMED tariff (expected) per person for GP consultations at canton level. Lines were fitted values from the mixed models. Marginal effects were shown. Dotted lines were borders of 95% confidence bands for the two groups.
(TIF)

S1 Dataset Number of GP consultations and relative costs in primary care for each canton and for each year from 2008 to 2014 and by patient’s age and sex. (XLSX)
