# Supplementary material for: Effects of a DRG-based hospital reimbursement on the health care utilization and costs in Swiss primary care: A retrospective “quasi-experimental” analysis
Source: PLoS One. 2020 Oct 27;15(10):e0241179. doi: 10.1371/journal.pone.0241179 (PMC7591068; doi:10.1371/journal.pone.0241179)
Supplement: S1 Checklist — (DOCX) [file pone.0241179.s001.docx]

**The RECORD statement – checklist of items, extended from the STROBE statement, that should be reported in observational studies using routinely collected health data.**

|  | **Item No.** | **STROBE items** | **Location in manuscript where items are reported** | **RECORD items** | **Location in manuscript where items are reported** |
| --- | --- | --- | --- | --- | --- |
| **Title and abstract** | | | | | |
|  | 1 | (a) Indicate the study’s design with a commonly used term in the title or the abstract (b) Provide in the abstract an informative and balanced summary of what was done and what was found | a)Title (A retrospective “quasi-experimental” analysis)  b)Abstract: Methods … Results | RECORD 1.1: The type of data used should be specified in the title or abstract. When possible, the name of the databases used should be included.  RECORD 1.2: If applicable, the geographic region and timeframe within which the study took place should be reported in the title or abstract.  RECORD 1.3: If linkage between databases was conducted for the study, this should be clearly stated in the title or abstract. | Abstract: Methods (retrospective analysis of data from health insurers)  Title (Swiss primary care);  Abstract (yearly data, from 2008 to 2014, of 60 Swiss health insurers. Patients represented 76% of the Swiss population)  (Only the number of inhabitants - Swiss population- were extracted to correct the outcomes: Abstract: Swiss population is mentioned) |
| **Introduction** | | | | | |
| Background rationale | 2 | Explain the scientific background and rationale for the investigation being reported | Lines 73-101 (In 2012, the DRG system, called Swiss Diagnosis Related Groups (Swiss DRG), was implemented in the whole country …) |  |  |
| Objectives | 3 | State specific objectives, including any prespecified hypotheses | Lines 99-101 (the aim of the current study is to better investigate the impact of SwissDRG on costs and volumes in the primary care sector, analyzing data, from 2008 to 2014, of all Swiss cantons.) |  |  |
| **Methods** | | | | | |
| Study Design | 4 | Present key elements of study design early in the paper | Lines 175-185 Study Design (The study was retrospective with a quasi-experimental fuzzy design …) |  |  |
| Setting | 5 | Describe the setting, locations, and relevant dates, including periods of recruitment, exposure, follow-up, and data collection | Lines 120-123 (only consultations provided by primary care physicians, in the period from 2008 to 2014, were considered) |  |  |
| Participants | 6 | *(a) Cohort study* - Give the eligibility criteria, and the sources and methods of selection of participants. Describe methods of follow-up  *Case-control study* - Give the eligibility criteria, and the sources and methods of case ascertainment and control selection. Give the rationale for the choice of cases and controls  *Cross-sectional study* - Give the eligibility criteria, and the sources and methods of selection of participants  *(b) Cohort study* - For matched studies, give matching criteria and number of exposed and unexposed  *Case-control study* - For matched studies, give matching criteria and the number of controls per case | Lines 134-144 (In the primary analysis, we included only the consultations of patients living in the same canton of the GP….) | RECORD 6.1: The methods of study population selection (such as codes or algorithms used to identify subjects) should be listed in detail. If this is not possible, an explanation should be provided.  RECORD 6.2: Any validation studies of the codes or algorithms used to select the population should be referenced. If validation was conducted for this study and not published elsewhere, detailed methods and results should be provided.  RECORD 6.3: If the study involved linkage of databases, consider use of a flow diagram or other graphical display to demonstrate the data linkage process, including the number of individuals with linked data at each stage. | Lines 113-130 (The dataset contained, in one file, the list of all Swiss ambulatory care providers and the area of their practice locations. Each provider was anonymously identified through a number (ZSR) and classified into the medical specialties, according to the Swiss Medical Association (FMH).) |
| Variables | 7 | Clearly define all outcomes, exposures, predictors, potential confounders, and effect modifiers. Give diagnostic criteria, if applicable. | Lines 145-165 (GP consultations were adjusted for the permanent resident population by canton, age-group, and sex….) | RECORD 7.1: A complete list of codes and algorithms used to classify exposures, outcomes, confounders, and effect modifiers should be provided. If these cannot be reported, an explanation should be provided. | Lines 145-165 |
| Data sources/ measurement | 8 | For each variable of interest, give sources of data and details of methods of assessment (measurement).  Describe comparability of assessment methods if there is more than one group | Lines 118-120  (Consultations and reimbursements were classified by patient’s gender, by patient’s age groups (5 years classes, from age 0 to 95) and by community of patient residence…) |  |  |
| Bias | 9 | Describe any efforts to address potential sources of bias | Lines 135-136 (As a sensitivity analysis, we considered all the consultations at GP cantonal level.)  Regression modeling addressing confounding, |  |  |
| Study size | 10 | Explain how the study size was arrived at | Lines 108-112 (patient data represented 76% of the Swiss population) |  |  |
| Quantitative variables | 11 | Explain how quantitative variables were handled in the analyses. If applicable, describe which groupings were chosen, and why | Lines 188-211 |  |  |
| Statistical methods | 12 | (a) Describe all statistical methods, including those used to control for confounding  (b) Describe any methods used to examine subgroups and interactions  (c) Explain how missing data were addressed  (d) *Cohort study* - If applicable, explain how loss to follow-up was addressed  *Case-control study* - If applicable, explain how matching of cases and controls was addressed  *Cross-sectional study* - If applicable, describe analytical methods taking account of sampling strategy  (e) Describe any sensitivity analyses | Lines 216-253 |  |  |
| Data access and cleaning methods |  | .. |  | RECORD 12.1: Authors should describe the extent to which the investigators had access to the database population used to create the study population.  RECORD 12.2: Authors should provide information on the data cleaning methods used in the study. | Lines 169 (License to access the study data was provided by SASIS AG)  Lines 129: SASIS data were grouped at cantonal level, |
| Linkage |  | .. |  | RECORD 12.3: State whether the study included person-level, institutional-level, or other data linkage across two or more databases. The methods of linkage and methods of linkage quality evaluation should be provided. | Lines 145-147 (data adjusted for the resident population) |
| **Results** | | | | | |
| Participants | 13 | (a) Report the numbers of individuals at each stage of the study (*e.g.*, numbers potentially eligible, examined for eligibility, confirmed eligible, included in the study, completing follow-up, and analysed)  (b) Give reasons for non-participation at each stage.  (c) Consider use of a flow diagram | Not applicable (but Fig1 represents the groups of cantons by AP-DRG) | RECORD 13.1: Describe in detail the selection of the persons included in the study (*i.e.,* study population selection) including filtering based on data quality, data availability and linkage. The selection of included persons can be described in the text and/or by means of the study flow diagram. |  |
| Descriptive data | 14 | (a) Give characteristics of study participants (*e.g.*, demographic, clinical, social) and information on exposures and potential confounders  (b) Indicate the number of participants with missing data for each variable of interest  (c) *Cohort study* - summarise follow-up time (*e.g.*, average and total amount) | Not applicable |  |  |
| Outcome data | 15 | *Cohort study* - Report numbers of outcome events or summary measures over time  *Case-control study* - Report numbers in each exposure category, or summary measures of exposure  *Cross-sectional study* - Report numbers of outcome events or summary measures | Table 1.a – Table 1.b Lines 257-322 |  |  |
| Main results | 16 | (a) Give unadjusted estimates and, if applicable, confounder-adjusted estimates and their precision (e.g., 95% confidence interval). Make clear which confounders were adjusted for and why they were included  (b) Report category boundaries when continuous variables were categorized  (c) If relevant, consider translating estimates of relative risk into absolute risk for a meaningful time period | Table 2- Lines 324-375, Fig2, Fig3 |  |  |
| Other analyses | 17 | Report other analyses done—e.g., analyses of subgroups and interactions, and sensitivity analyses | S1 Fig, S1 Table |  |  |
| **Discussion** | | | | | |
| Key results | 18 | Summarise key results with reference to study objectives | Lines 399-439 |  |  |
| Limitations | 19 | Discuss limitations of the study, taking into account sources of potential bias or imprecision. Discuss both direction and magnitude of any potential bias | Lines 443-466 | RECORD 19.1: Discuss the implications of using data that were not created or collected to answer the specific research question(s). Include discussion of misclassification bias, unmeasured confounding, missing data, and changing eligibility over time, as they pertain to the study being reported. | Lines 444-448 (we did not include other relevant characteristics affecting GP consultations and costs;, we did not consider any other confounding factors -i.e., socio-economic characteristics)  Lines 457-458 (we have no data on inpatient-outpatient transitions) |
| Interpretation | 20 | Give a cautious overall interpretation of results considering objectives, limitations, multiplicity of analyses, results from similar studies, and other relevant evidence | Lines 401-414  Lines 421-439 |  |  |
| Generalisability | 21 | Discuss the generalisability (external validity) of the study results | Lines 472-479 |  |  |
| **Other Information** | | | | | |
| Funding | 22 | Give the source of funding and the role of the funders for the present study and, if applicable, for the original study on which the present article is based | Not Applicable |  |  |
| Accessibility of protocol, raw data, and programming code |  | .. |  | RECORD 22.1: Authors should provide information on how to access any supplemental information such as the study protocol, raw data, or programming code. | Data Availability (We uploaded the minimal anonymized data set necessary to replicate our study findings) |

*Reference: Benchimol EI, Smeeth L, Guttmann A, Harron K, Moher D, Petersen I, Sørensen HT, von Elm E, Langan SM, the RECORD Working Committee. The REporting of studies Conducted using Observational Routinely-collected health Data (RECORD) Statement. *PLoS Medicine* 2015; in press.

*Checklist is protected under Creative Commons Attribution ([CC BY](http://creativecommons.org/licenses/by/4.0/)) license.
