# Supplementary figures and images for: Effects of a DRG-based hospital reimbursement on the health care utilization and costs in Swiss primary care: A retrospective “quasi-experimental” analysis
Source: PLoS One. 2020 Oct 27;15(10):e0241179. doi: 10.1371/journal.pone.0241179 (PMC7591068; doi:10.1371/journal.pone.0241179)

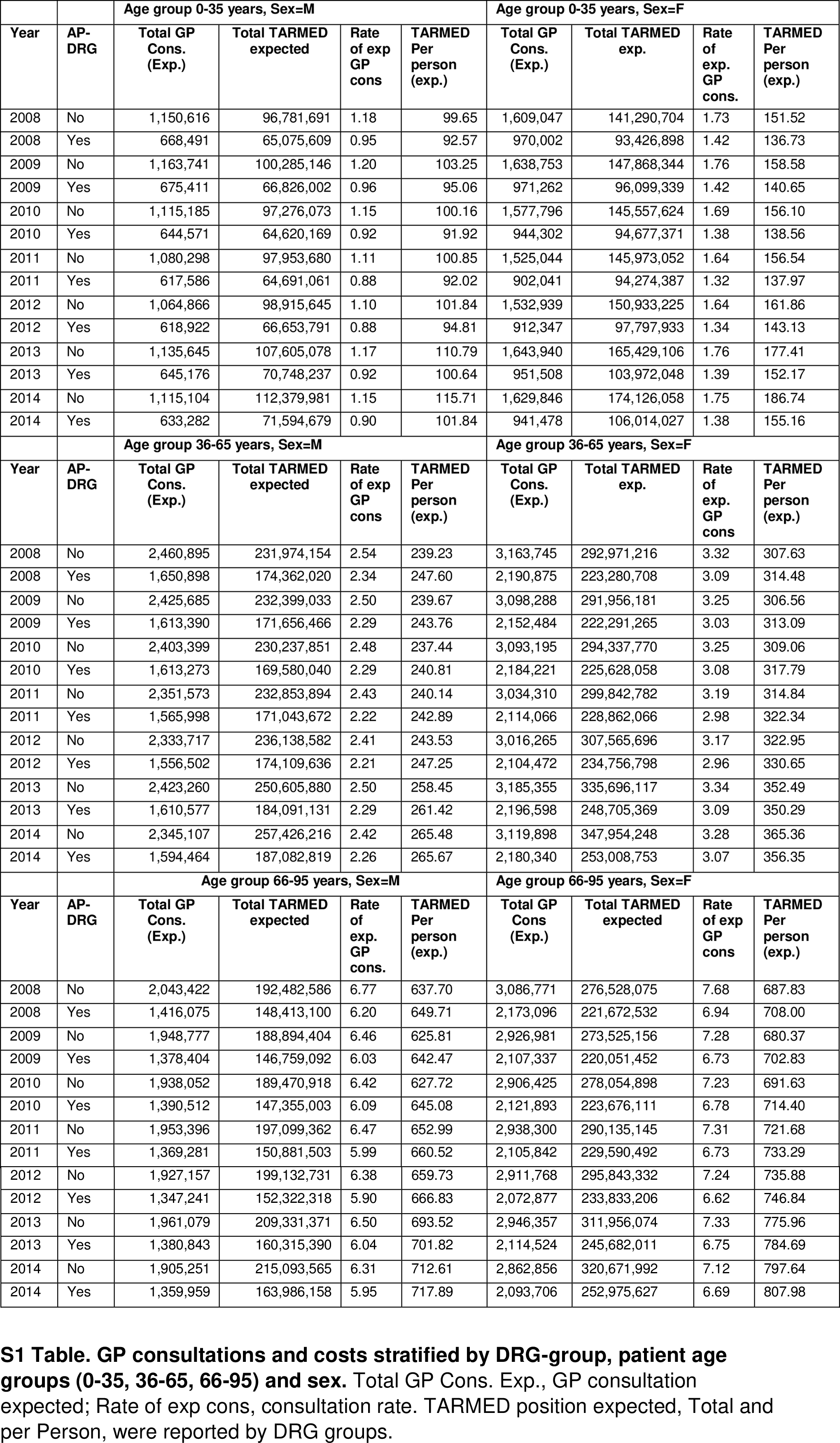

Supplement: S1 Table — Total GP Cons. Exp., GP consultation expected; Rate of exp cons, consultation rate. TARMED position expected, Total and per Person, were reported by DRG groups. (TIF) [file pone.0241179.s002.tif]

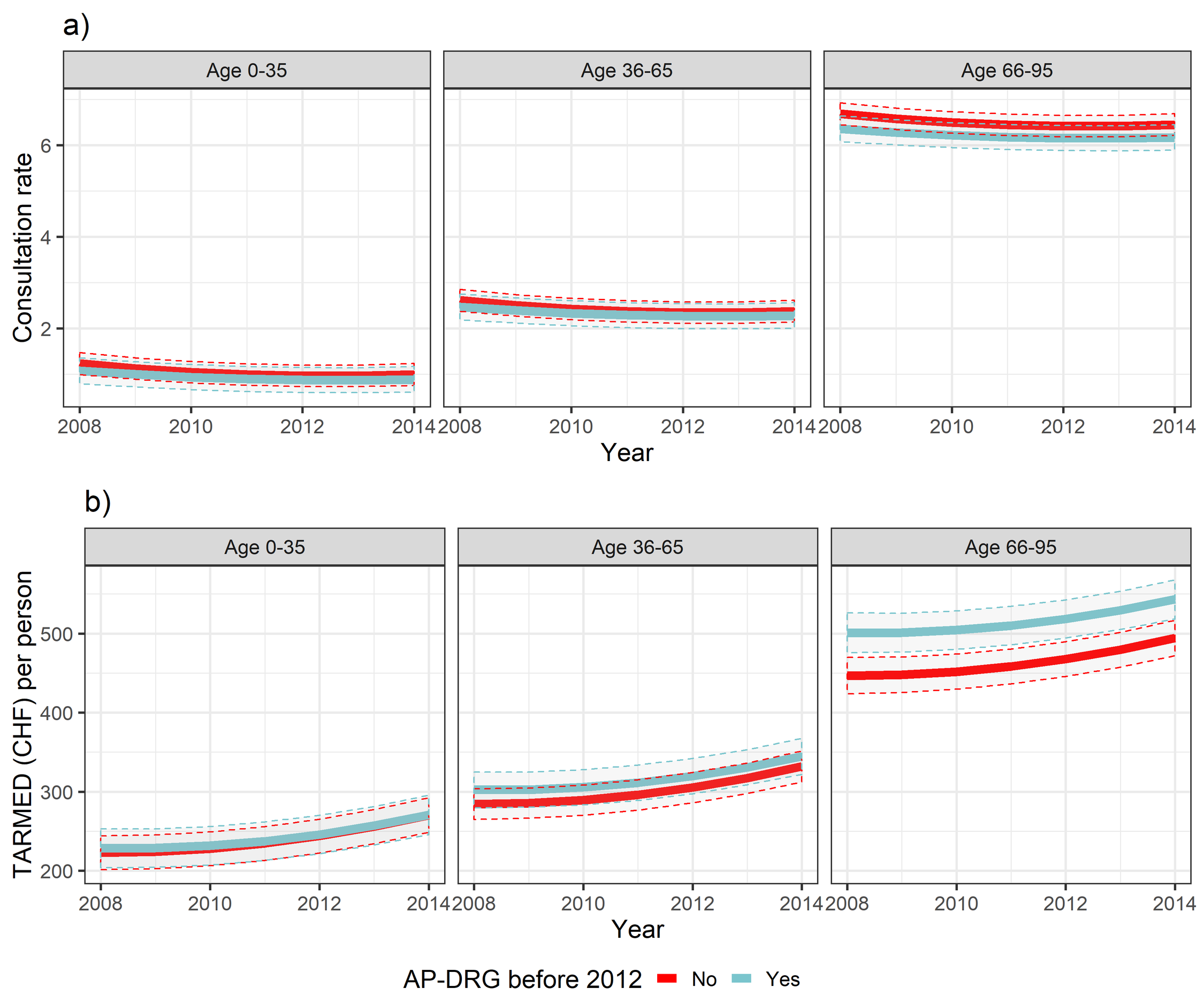

Supplement: S1 Fig — Effects of DRG over time and for patient’s age groups on: a) Rate of expected GP consultations; b) TARMED tariff (expected) per person for GP consultations at canton level. Lines were fitted values from the mixed models. Marginal effects were shown. Dotted lines were borders of 95% confidence bands for the two groups. (TIF) [file pone.0241179.s003.tif]
